# Independent concentration extraction as a novel green approach resolving overlapped UV–Vis binary spectra and HPTLC-densitometric methods for concurrent determination of levocloperastine and chlorpheniramine

**DOI:** 10.1186/s13065-024-01260-w

**Published:** 2024-08-28

**Authors:** Ekram H. Mohamed, Hany A. Batakoushy, Adel Ehab Ibrahim, Zeinab Adel Nasr, Marwa M. Soliman, Sona S. Barghash, Tahany F. Mohamed, Fatma A. Fouad

**Affiliations:** 1 Pharmaceutical Analytical Chemistry Department, Faculty of Pharmacy, The British University in Egypt, El Sherouk City, 11837 Egypt; 2 Pharmaceutical Analytical Chemistry Department, Faculty of Pharmacy, Menoufia University, Shebin Elkom, 32511 Egypt; 3 Pharmaceutical Analytical Chemistry Department, Faculty of Pharmacy, Port-Said University, Port Said, 42511 Egypt; 4 Natural and Medical Sciences Research Center, University of Nizwa, Birkat Al Mauz, P.O. Box 33, Nizwa, 616 Oman; 5 Pharmaceutical Analytical Chemistry Department, Faculty of Pharmacy (Girls), Al-Azhar University, Cairo, 11754 Egypt

**Keywords:** Green analytical chemistry, Spectral resolution, Independent concentration extraction, High performance thin layer chromatography, Chlorpheniramine, Levocloperastine

## Abstract

**Background:**

The proposed research study introduces independent concentration extraction (ICE) as a novel UV–Vis spectrophotometric approach. The approach can be used for extracting the concentration of two analytes with severely overlapped spectra from their binary mixtures. ICE is based on spectral extraction platform involving simple smart successive methods that can directly extract the original zero order spectra of the analytes at their characteristic (λ_max_). Chlorpheniramine maleate (CPM) and Levocloperastine fendizoate (LCF) are two commonly co-formulated drugs in cough preparations. The combined mixture was used to confirm the validity of the developed ICE tool. Another less green HPTLC was developed for the first time to separate both drugs and help also in confirming the proposed tool.

**Methods:**

For the simultaneous determination of CPM and LCF, two ecologically friendly techniques were employed. The first approach encompasses the use of the ICE spectrophotometric method that could be successively applied for extracting the concentration of two analytes with severely overlapped unresolved spectra in their binary mixtures. Other complementary methods aiming at original spectral extraction; including spectrum subtraction (SS) and unity subtraction (US) were also successfully employed to resolve the zero order spectra of the combined drugs with all their characteristic features and peaks. The second technique used, a high-performance TLC-densitometric one, was performed on silica plates with silica plates F254 and a mobile phase with a ratio of 3:3:3:1 by volume of toluene, ethanol, acetone, and ammonia as a developing system at 230 nm.

**Results:**

The presented extraction approach was executed without any optimization steps or sample pretreatment for the simultaneous determination of CPM and LCF. The method was found to be valid for their determination within concentration range of 3.0–30.0 μg mL^−1^ for both drugs. For HPTLC method, the resulting R_f_ values of CPM and LCF were 0.37 and 0.78, within concentration ranges of 0.3–4.0 μg/spot and 0.8–10.0 μg/spot, respectively. Greenness assessment of both developed methodologies showed that the HPTLC method is less green than the spectrophotometric method, yet with comparable sustainability when it comes to the used technique.

**Conclusion:**

The procedures were found to be selective, accurate, and precise for analysis of the studied binary mixture. Furthermore, the environmental impact of the introduced methods was assessed using novel greenness metrics, namely AGREE and Green Analytical Procedure Index (GAPI) to prove their ecological safety. In addition, white analytical chemistry (WAC) evaluation metric was employed to ensure the synergy and coherence of analytical, practical, and ecological attributes.

**Supplementary Information:**

The online version contains supplementary material available at 10.1186/s13065-024-01260-w.

## Background

The importance of pharmaceutical analysis (PA) is increasing due to the globalization of pharmaceutical market and enhanced concurrence between manufacturing companies where it is directly related to drug efficacy and safety. The main aim of PA is to develop, apply and validate the good manufacturing practices (GMP) to assure the production of safe pharmaceutical dosage forms and products of standard high quality. This could be achieved via tracking the concentration of active pharmaceutical ingredients throughout the whole manufacturing steps starting from purchasing the bulk powder or its raw materials passing through the research and developments stages. Various instrumental methods of analysis could be used in PA. the selection of the optimum method of analysis depend on many factors including the nature of compound under investigation, the complexity of the mixture, the underlying matrix, sample size, as well as the analysis purpose [1].

Optical methods in general and UV–VIS spectroscopy in specific are the most convenient technique used for routine analysis of active pharmaceutical ingredients either due to their simplicity, cost effectiveness and the availability of its appliances in nearly all quality control laboratories. Moreover, UV spectroscopy methods are also considered to be ecofriendly where safe solvents are usually used as water, ethanol or methanol beside low wastes are generated. The specificity of the UV spectroscopic method was greatly improved by the introduction of smart resolution techniques [2–4], that enabled the determination of multicomponent complex mixtures using simple mathematical concepts without the need for any sophisticated or expensive instruments or programs.

Meanwhile, high performance thin layer chromatography (HPTLC) has gained a great momentum during the past few decades especially with the advanced densitometric detection which is a more advanced and improved version of the traditional TLC. HPTLC offers higher separation efficiency, improved resolution, and better reproducibility [5]. The application of HPTLC-densitometric technique was found to give fast and accurate results while using very small sample amounts. In addition, HPTLC-densitometric approach is cheap and doesn’t need complicated procedures or instrumentation as those required by HPLC [6].

**Fig. 1:**
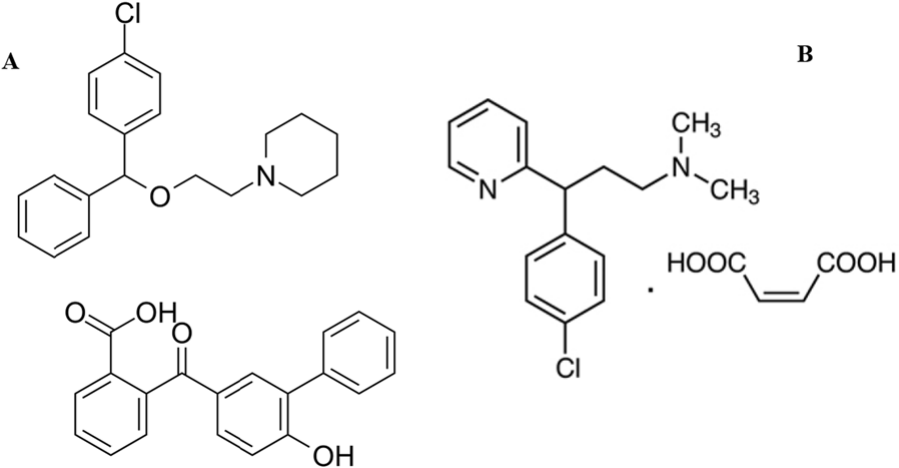
Chemical structure of Levochlopersatine Fenodizoate (**A**) and Chlorpheniramine Maleate (**B**)

Cough is a reflex action that helps to clear the throat and airways of irritants, mucus, or foreign substances. Cough could be brought on by acute and chronic respiratory disorders, as well as allergic conditions and asthma. The body uses coughing, whether it is productive or dry, to remove irritants from the airways and guard against infection [7]. Several medication combinations are frequently used to treat coughs [8, 9]. Chlorpheniramine maleate (CPM; chemical structure Fig. 1) is one of the most commonly used medications for the treatment of allergic diseases [9]. Levocloperastine fendizoate (LCF; chemical structure Fig. 1) is acting centrally as a cough suppressant [10]. Combination of both medications that is primarily used to treat dry cough [11, 12]. In such combination, CPM blocks the action of histamine, a chemical that triggers allergic reactions, and helps to relieve allergy symptoms like itching, swelling, congestion, and stiffness. Meanwhile, LCF blocks the transmission of nerve signals from the cough center in the brain to the muscles that cause coughing [7].

Reviewing literature showed various techniques that had been developed to analyze CPM and LCF separately [13] or in their combination with other drugs [9, 14]. For the simultaneous analysis of both drugs, only few HPLC methods have been reported [15, 16]. To our knowledge, no UV–Vis spectrophotometry nor TLC-densitometry methods have been yet reported for the simultaneous analysis of the drugs under study. The main objectives of this work are, firstly, to establish ICE approach and confirm its validation via simple eco-friendly steps for the simultaneous determination of CPM and LCF in bulk powder or in their combined dosage form. Another aim is to develop a novel green and sustainable HPTLC-densitometry method that could concurrently estimate CPM and LCF. Moreover, the suggested methods were assessed for their greenness using different tools including the green analytical procedure index (GAPI) [17], AGREE [18], and the white analytical chemistry [19] metrics which showed that the proposed approaches have high sustainability.

## Theoretical concept for the novel spectrophotometric approach

### Independent concentration extraction (ICE)

Independent Concentration extraction (ICE) is a new simple method applied for the first time for the direct estimation of analytes of interest with no need for any other complementary method. It should be considered to be an extension to the previously applied constant extraction (CE) method for determination of binary mixture with overlapping [20]. The main difference between CE and the proposed ICE is the use of the normalized spectra of drug of interest as a divisor. Thus, modulating the extracted constant directly to the concentration [21–23], with no need to the constant multiplication step found in CE method. The novel ICE method could be used to quantify two components (e.g., M and N) with complete spectral overlap via only one main step depending on complementary and successive mathematical equations as summarized in the following scheme.

For determination of the first drug (N), as a divisor, the spectrum of the total binary mixture is divided by the normalized spectra of M (${ns}_{N}$). The obtained ratio spectrum could be expressed as following.

1 $$M + N \div ns_{N} = \frac{M}{{ns_{N} }} + \frac{N}{{ns_{N} }}$$

2 $$M + N \div ns_{N} = \frac{M}{{ns_{N} }} + concentratin\;of\;N$$

The ratio spectrum’s amplitude is measured at two different wavelengths (λ1 and λ2), where the two drugs overlap. The amplitude difference (ΔP_mix_) is then computed where the constant value resembling the concentration of N would be cancelled and Δ P_mix_ is directly related to component M only as summarized in the following equation.

3 $$\Delta {\text{P}}_{{{\text{mix}}}} = {\text{P}}\lambda {1} - {\text{P}}\lambda {2} = \left( {\left( {\frac{M}{{ns_{N} }}} \right)\lambda 1 + concentratin\;of\;N} \right)1 - \left( {\left( {\frac{M}{{ns_{N} }}} \right)\lambda 2 + concentratin\;of\;N} \right)2$$

4 $$\Delta {\text{P}}_{{{\text{mix}}}} = \left( {\frac{M}{{ns_{N} }}} \right)\lambda 1 - \left( {\frac{M}{{ns_{N} }}} \right)\lambda 2$$

The same amplitudes previously recorded at the selected wavelengths (λ1 and λ2) are summed and the summation value ($\sum {{\text{P}}_{{{\text{mix}}}} }$) is presented in the following equation.

5 $$\sum {{\text{P}}_{{{\text{mix}}}} = {\text{P}}\lambda {1} + {\text{P}}\lambda {2} = \left( {\left( {\frac{M}{{ns_{N} }}} \right)\lambda 1 + concentratin\;of\;N} \right)1 + \left( {\left( {\frac{M}{{ns_{N} }}} \right)\lambda 2 + concentratin\;of\;N} \right)2}$$

6 $$\sum {{\text{P}}_{{{\text{mix}}}} = \left( {\left( {\frac{M}{{ns_{N} }}} \right)\lambda 1} \right) + \left( {\left( {\frac{M}{{ns_{N} }}} \right)\lambda 2} \right) + 2\;concentratin\;of\;N}$$

Using the normalized spectrum of N (ns_N_) as a divisor for different concentrations of M in its pure form, a direct correlation is established between the amplitude difference (ΔP_M_) at the two preselected wavelengths and the summed or total amplitude at the same wavelengths (P_M Sum_) and the regression equation is expressed as follows.

7 $$\Delta {\text{P}}_{{\text{M}}} = {\text{Slope}}\;{\text{P}}_{{{\text{M}}\;{\text{Sum}}}} + {\text{intercept}}$$

8 $$\left( {\frac{M}{{ns_{N} }}} \right)\lambda 1 - \left( {\frac{M}{{ns_{N} }}} \right)\lambda 2 = {\text{Slope }}\left( {\left( {\frac{M}{{ns_{N} }}} \right)\lambda 1 + \left( {\frac{M}{{ns_{N} }}} \right)\lambda 2 + } \right. intercept$$

To calculate the concentration of component N, the amplitude values difference (ΔP_mix_) and sum (∑P_mix_) at the two selected wavelengths on the ratio spectra, obtained by dividing the total spectrum of the binary mixture by ns N, were calculated as presented in Eqs. (4) and (6), respectively. The same calculated amplitude difference was further manipulated to compute the (P_M_ Sum) of pure component M from the corresponding regression Eq. (7).

The obtained (P_M_ Sum) for pure M is then subtracted from the (∑P_mix_) and the concentration of N multiplied by 2 as summarized in the following equation.

9 $$\sum {\text{P}}_{{{\text{mix}}}} - {\text{P}}_{{\text{M}}} {\text{Sum}} = \left( {\left( {\frac{M}{{ns_{N} }}} \right)\lambda 1} \right) + \left( {\left( {\frac{M}{{ns_{N} }}} \right)\lambda 2} \right) + 2\;concentratin\;of\;N - \left( {\frac{M}{{ns_{N} }}} \right)\lambda 1 + \left( {\frac{M}{{ns_{N} }}} \right)\lambda 2$$

10 $$\sum {\text{P}}_{{{\text{mix}}}} - {\text{P}}_{{\text{M}}} {\text{Sum}} = 2\;concentratin\;of\;N$$

The concentration of N could be directly obtained by dividing Eq. (10) by 2.

For determination the concentration of the second component M, the same procedures should be repeated but with using the normalized spectrum of M (ns M) as divisor in all the previous steps instead of (ns N) or using Amplitude Difference method [24].

After estimation of the first component using ICE method, the second drug in the binary mixture could be also determined via other independent zero order recovering methods including Zero Order Extraction and Unity Subtraction method [25].

### Zero order extraction method (Z°E)

The method aims at obtaining the original zero order spectrum of the compound under study which is considered to be a unique fingerprint for each compound and hence enabling its estimation at the wavelength of maximum absorption (λmax). The method could be easily performed through two simple steps. The first step is multiplying the obtained concentration of N, via ICE method, by its normalized spectrum to recover the whole zero order spectrum of N (D° N) in the binary mixture [21, 23]. The (D° N) is then subtracted from the total spectrum of the binary mixture (D° mix) to finally extract the zero-order spectrum of M (D° M) actually found in the mixture. The concentration of M is determined through a correlation between the absorbance of different concentration at its λmax and the respective concentrations. ZE method is an independent method and could only be performed after quantification of the first component of the mixture as summarized in the following two steps.

11 $${\text{The}}\;{\text{Concentration}}\;{\text{multiplication}}\;{\text{step: Concentration}}\;{\text{of}}\;{\text{N}} \times {\text{ns}}\;{\text{N}} = {\text{D}}^\circ \;{\text{N}}$$

12 $${\text{The}}\;{\text{Absorption}}\;{\text{Subtraction}}\;{\text{step: D}}^\circ \;{\text{mix}} - {\text{D}}^\circ \;{\text{N}} = {\text{D}}^\circ \;{\text{M}}$$

### Unity subtraction method (US)

The unity subtraction method (US) also known as unified constant subtraction is one of fingerprint resolving technique previously reported [25]. It could be adopted for resolution of zero order spectrum of the second component in the binary mixture after estimation of the first via applying successive spectral manipulation steps. US method starts by multiplying the obtained N concentration in the ICE to extract its zero-order spectrum D° N in the binary mixture as mentioned in Eq. (11). The extracted D° N is used as a divisor for the whole spectrum of the binary mixture as follows.

13 $$\frac{D^\circ \;mix}{{D^\circ \;N}} = \frac{D^\circ \;M}{{D^\circ \;N}} + \frac{D^\circ \;N}{{D^\circ \;N}}$$

14 $$\frac{D^\circ \;mix}{{D^\circ \;N}} = \frac{D^\circ \;M}{{D^\circ \;N}} + 1$$

By subtracting the constant value 1 from the obtained ratio spectrum $\frac{D^\circ mix}{D^\circ N}$ and then multiplying by the same divisor used (D° N), the original zero order spectrum of M (D° M) will be resolved and directly determined at its λmax as summarized in brief.

15 $$\frac{D^\circ \;mix}{{D^\circ \;N}} - 1 = \frac{D^\circ \;M}{{D^\circ \;N}} + 1 - 1$$

16 $$\frac{D^\circ \;mix}{{D^\circ \;N}} - 1 = \frac{D^\circ \;M}{{D^\circ \;N}} \times {\text{D}}^\circ \;{\text{N}}$$

17 $$\frac{D^\circ \;mix}{{D^\circ \;N}} - 1 = {\text{D}}^\circ \;{\text{M}}$$

## Experimental

### Apparatus

Shimadzu’s UV–Vis 1601 PC spectrophotometer (Tokyo, Japan) was used for the spectral analysis. Zero order spectra of the prepared solution were recorded over a wavelength range of 200 to 400 nm.

TLC chromatography was performed on precoated TLC plates (20 × 20 cm, 0.22 mm thickness) using silica gel 60 GF254 from Merck (Darmstadt, Germany). For data collection, a Linomat 5 auto sampler and scanner from Camag (Muttenz, Switzerland) was utilized. To apply the sample, a Hamilton 100 μL syringe (Bonaduz, Switzerland) was used. The scanning mode was absorbance, and the slit dimension was 3 mm × 0.45 mm with a scanning speed of 20 mm s^−1^.

### Materials and reagents

Chemi-Pharm Pharmaceutical Company (Cairo, Egypt) generously provided LCF, while Memphis Pharmaceutical Company (Cairo, Egypt) generously provided CPM. For CPM and LCF, the purity was reported to be (99.89%) and (99.92%), respectively. Ethanol was of analytical grade and was purchased from Sigma-Aldrich (Darmstadt, Germany). El-Nasr Pharmaceutical Company (Cairo, Egypt) supplied analytical grades of toluene and ammonia (25%, v/v).

Lupituss-CPM® syrup (Lot number: M181259), containing 20 mg LCF and 4 mg CPM per 5 mL, was purchased from Lupin Ltd. (Mumbai, India).

### Standard solutions and laboratory prepared mixtures

In order to prepare separate stock standard solutions of CPM and LCF (1 mg mL^−1^), ethanol was used as a solvent. Working solutions were then prepared by appropriately diluting each stock solution with ethanol to get 0.1 mg mL^−1^ of each drug concentration. Transferring aliquots from the drugs working solutions (0.1 mg mL^−1^), mixing them thoroughly, and filtering them before adding ethanol to volume allowed to create mixtures at various ratios for CPM and LCF. For spectrophotometric methods, the final ratios of both drugs were (1:1, 1:2, 1:3, 1:5, 2:1, 3:1, 4:1 and 5:1, CPM: LCF, respectively).

### Spectroscopy procedures

#### Scanning of the zero order spectra

**Fig. 2:**
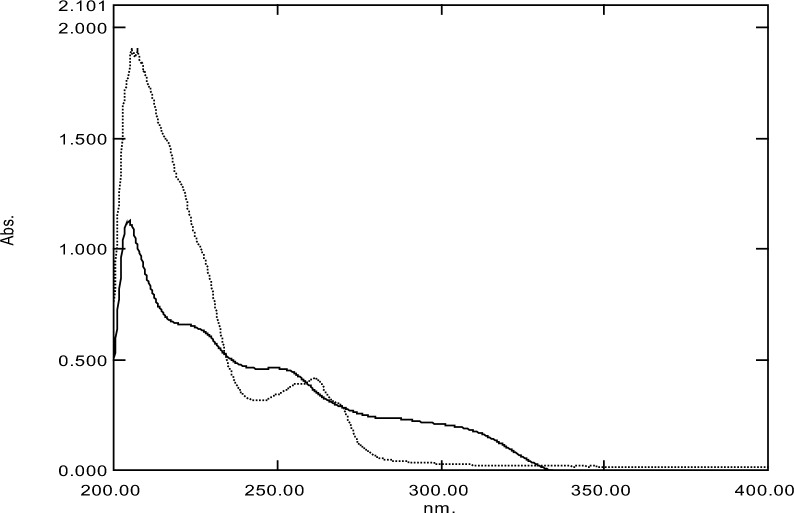
Zero order spectra of 10 μg mL^−1^ of CPM (―) and LCF (……)

The spectrum of drugs under study using concentration of 10 μg mL^−1^ for both were separately scanned within 200–400 nm wavelength range and intervals of 0.1 nm intervals. The scanned spectra were recorded and stored with the aid of the spectrophotometer software as displayed in Fig. 2. The spectra of both drugs were overlaid and a sever overlap was observed with along the whole wavelength range with no drug extended over the other hindering their direct determination. New smart spectrophotometric method based on simple mathematical approaches could be simply used for resolving tangled spectra.

#### Preparation of calibration set and preparation of normalized spectrum of LCF

Seven different concentrations were accurately prepared by transferring different aliquots equivalent to 30–300 μg of both CPM and LCF from their corresponding working solutions into two sets of 10-mL volumetric flasks. The volume was completed to the mark using ethanol as a solvent. The spectra of different concentrations of both drugs were scanned within the 200–400 nm range using the same solvent as the blank and saved using the spectrophotometer software. For the normalized spectrum of LCF; the stored spectra of different concentrations of LCF were recalled and separately divided by the respective concentration and an average spectrum (1 μg mL^−1^) was recorded and stored.

## Construction of calibration graphs

### Independent concentration extraction (ICE) method

Solutions of different concentrations of pure CPM within 2–30 μg mL^−1^ were separately divided by the normalized spectrum of LCF. The amplitude difference and summation at 263 nm and 300 nm were calculated for each concentration. A calibration curve was constructed relating the amplitude difference values against the amplitude summation values and the regression equation was computed.

### Zero order extraction (Z°E) and unity subtraction methods (US)

Two separate calibration curves were constructed; the first one represented a correlation between the absorbance values of different LCF standard solutions at its λ_max_ 250.6 nm against the corresponding concentration. The second calibration graph is constructed between the absorbance values at λ_max_ 261.6 nm for different standard solutions of CPM and corresponding concentrations. The respective regression equation for each of CPM and LCF was computed to be used for calculating unknown concentrations for both drugs respectively.

## Analysis of laboratory prepared mixtures

To apply the ICE method, The D^0^ of the synthetic binary mixtures previously prepared was separately divided by the normalized spectrum of LCF. A new ratio spectrum was obtained for each mixture where the amplitude values at 263 nm and 300 nm were recorded. The amplitude difference (ΔP mix) and amplitude summation (∑P mix) were calculated for each mixture. The amplitude difference values were further manipulated to compute the unknown amplitude summation value (P_CPM_ sum) of standard CPM using the corresponding regression equation. The (P_CPM_ sum) is then subtracted from the total (∑P mix) to obtain a constant value resembling the concentration of LCF in the mixture multiplied by 2 (2 × C_LCF_). The constant is then divided by 2 to obtain the concentration of LCF. The same steps could be repeated to obtain the concentration of CPM but using its normalized spectrum instead of that of LCF as a divisor, but for simplification ICE was coupled with Z°E and US to estimate the concentration of CPM at its λ_max_ using the corresponding regression equation.

### TLC procedures

#### Optimization of chromatographic conditions for HPTLC-densitometry

The analysis was performed on pre-coated 20 × 10 cm TLC aluminium silica gel 60 GF254 plates. An amount of 10 μL samples were applied to the plates at 3 mm band width and at intermittent spaces of 10 mm between bands. The injection was performed at 1 cm distance from the bottom edge. The mobile phase was used to pre-saturate the chromatographic chamber for 20 min before developing it in an ascending fashion with toluene: As the mobile phase, ethanol: acetone: ammonia (3:3:3:1, by volume) was used. Separate aliquots of both drugs' working solutions were applied to the TLC plates in triplicate, which were then air dried at room temperature before being scanned at 230 nm with a CAMAG TLC scanner.

#### Construction of calibration graphs

To obtain final concentrations of 0.3–4.0 μg/spot for CPM and 0.8–10 μg/spot for LCF, aliquots from the working standard solutions of CPM and LCF were accurately measured and separately applied to TLC plates in triplicates. An aliquot of 10 μL of each solution was applied to pre-washed activated plates, and the plates were developed with a mobile phase of toluene, ethanol, acetone, and ammonia acid (3:3:3:1 by volume). The appropriate drug concentration was then plotted against the peak area to generate a regression equation.

### Analysis of laboratory prepared mixtures

Solutions containing the above-mentioned ratios of CPM and LCF were placed into 10 mL volumetric flasks, and the volume was then completed with ethanol. The solutions were then analyzed using the chromatographic procedures previously described and the obtained regression equations were used to compute the % found for each drug.

### Analysis of pharmaceutical formulations

In a 100-mL volumetric flask, 25 mL of Lupituss-CPM® Syrup, equivalent to 20 mg of CPM and 100 mg of LCF, were dissolved in 25 mL of ethanol to obtain concentrations of 0.2 and 1.0 mg mL^−1^ for CPM and LCF, respectively. Different concentrations were analysed in the manner described under calibration curve construction for both techniques, and the concentrations of both drugs were calculated.

## Results and discussion

### Extraction approach

The overlaid spectra of CPM and LCF exhibited severe overlap along the whole wavelength range selected for measurement. No zero crossing points or extension of one drug over the other were displayed hindering the direct estimation of the drugs. A new spectrophotometric platform based on extraction methodology was suggested for the resolution of CPM and LCF and binary mixture. The technique involved three different methods. The first one is the independent concentration extraction (ICE) method could directly extract the concentration of both components consequently with no need for any other complementary method or any specific prerequisite such as presence of isosbestic point, dual wavelength, or extension of one component over the other**.** The only prerequisite to execute the method is preparing and recording the normalized spectrum of the drug of interest. The other two methods namely Zero Order Extraction (Z°E) and Unity Subtraction (US) methods are dependent methods used for determination of the second component following few simple steps but need to be coupled with other methods for determination of the first drug.

The three proposed methods lie under the umbrella of spectrophotometric extracting technique where they extract important hidden features from the overlapped spectra of CPM and LCF. The ICE directly extracts the concentration of the main or first component using its normalized spectrum as a divisor and upon its coupling with either Z°E or US methods, the whole zero order spectrum of both components could be extracted with all their inherent features and peaks enabling their accurate estimation at their λ_max_. The order spectrum of any component is also considered to be a fingerprint and plays a role in determination of the purity index.

### Independent concentration extraction (ICE)

**Fig. 3:**
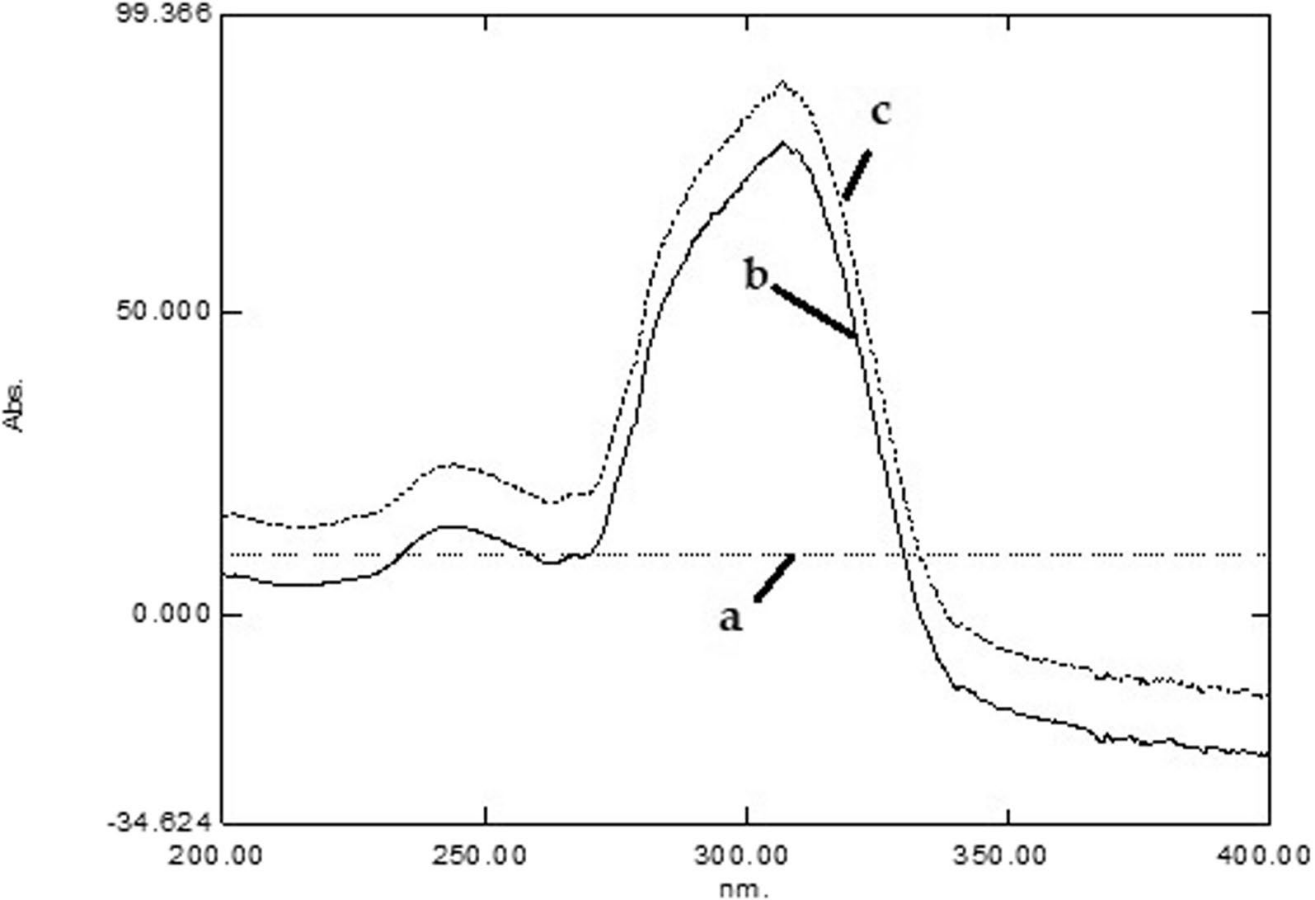
UV/VIS normalized spectra of LCF (**a**; 10 µg mL^−1^), CPM (**b**; 10 µg mL^−1^) and binary mixture of LCF/CPM (**c**; 10 µg mL^−1^, each). The normalization method was achieved by dividing the measured spectra by that of a reference of LCF (concentration 1 µg mL^−1^)

ICE starts by the dividing the binary mixture either synthetically prepared in the laboratory or in tablet dosage form by the stored normalized spectrum of LCF where a new ratio spectrum (CPM + LCF)/ns LCF that could also be expressed as (CPM/ LCF + concentration of LCF) as displayed in Fig. 3. As shown in Fig. 3, three normalized spectra appear. The first one (Fig. 3a) represents a constant value obtained by dividing the original spectrum of LCF (10 µg mL^−1^) by the reference divisor spectrum (LCF, 1.0 µg mL^−1^) which was represented as a straight line parallel to the origin. The other 2 normalized spectra represent the normalized spectra of CPM alone (Fig. 3b) and CPM/LCF mixture (Fig. 3c) at the same concentrations (10 µg mL^−1^).

Two wavelengths with significantly different amplitudes were selected carefully on the above ratio spectra (263 nm and 300 nm). The amplitude at the chosen wavelengths was subtracted one time to get the amplitude difference (ΔP) and added to get the summed amplitude (∑P) another time. The ΔP was further manipulated to calculate the summed of pure LCF from the previously computed corresponding regression equation amplitude (P_LCF_ Sum). Upon subtracting P_LCF_ Sum from the total ∑P, the result is a constant representing double the concentration of LCF. Hence, the concentration of LCF in the mixture could be obtained by dividing the constant by 2. The same steps could be followed for direct determination of CPM concentration using its normalized spectrum as the divisor instead. To avoid repetition and to display the extraction technique, which is the main aim, the ICE method was only used for determination of LCF and coupled with other extracting methods for determination of CPM.

### Zero order extraction method (Z°E)

The Zero Order Extraction method could also be applied for determination of CPM concentration via extracting its zero order absorption spectra. The concentration of LCF, obtained using Independent Concentration Extraction, was multiplied by its normalized spectra (ns LCF) to get the zero-absorption spectrum of LCF (D°LCF) originally present the binary mixture. Finally, the zero-order absorption spectrum (CPM) could be easily extracted by direct subtraction of the D°LCF from the total absorption spectrum of the binary mixture. The absorption value at its λ_max_ (261.6 nm) was recorded and used to calculate the concentration using the corresponding regression equation.

### Unity subtraction (US) method

Another facile approach to determine the concentration of CPM is the Unity subtraction method in which the exact (D° LCF) obtained via applying ICE method, as detailed under (3.1), was used as a divisor. Upon dividing the zero-order spectrum of the binary mixture by the (D° LCF) having the same concentration of LCF present the mixture, a ratio spectrum was obtained ($\frac{D^\circ CPM}{D^\circ LCF}+1$). To get the zero-order spectrum of CPM, subtract number (1) from the above ratio spectrum and then multiply the resulted spectrum ($\frac{D^\circ CPM}{D^\circ LCF}$) by the used divisor to finally obtain $D^\circ CPM$.

### TLC-densitometric method

The proposed method was based on the difference in Rf values [26] between CPM and LCF, which results from differences in their polarities and migration rates on silica plates. To achieve the best separation of the two drugs, different solvent systems were used to optimise the chromatographic conditions. In terms of mobile phase selection and optimization, efforts had been made to achieve an eco-friendly solvent system without sacrificing the analytical efficiency.

**Fig. 4:**
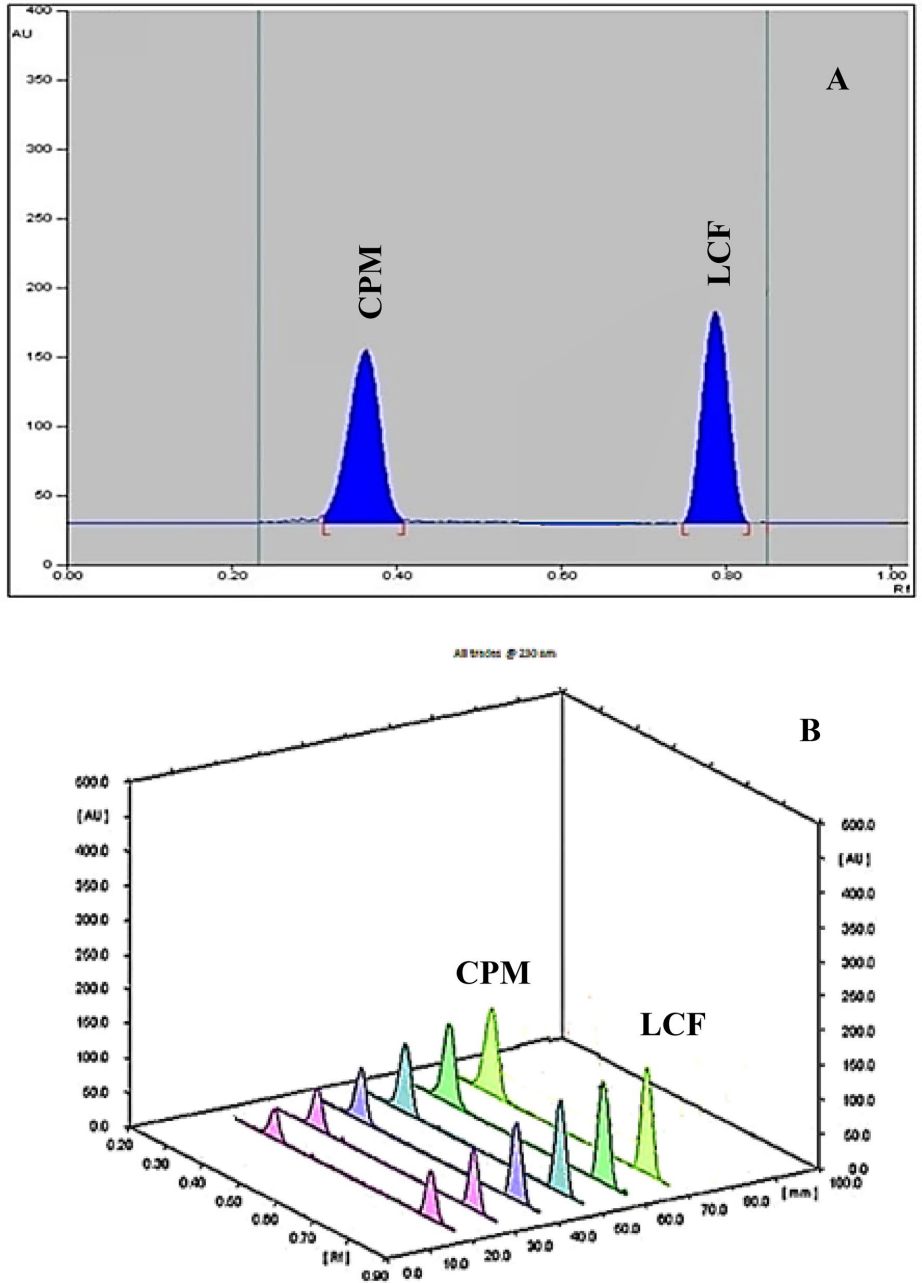
Two-dimensional (**A**) and three-dimensional (**B**) HPTLC densitogram of (4.0 μg/spot) of CPM and LCF at 230 nm, detection wavelength

Finding the best solvent system is usually the most difficult part of developing a TLC method, especially if the mobile phase has never been reported before, as in our case. Although starting with a large proportion of nonpolar solvent, such as chloroform, hexane, or benzene, is a common method for achieving good separation. Because of their well-known environmental hazards, these solvents were excluded from our trials. Several trials were conducted using various solvent systems, including ethanol, acetone, and ethyl acetate in various ratios (2:4:4, 6:2:2, and 4:3:3 by volume), but no separation was obtained, even after modifying the pH with formic acid, acetic acid, or ammonia. Other systems were tested, including toluene: ethanol: acetone in various ratios (2:2:6, 4:2:4, 3:3:4), with the toluene: ethanol: acetone (3:3:4 by volume) system demonstrating good separation but with tailing of the two drugs. As a result, we experimented with different pH levels by adding either ammonia or glacial acetic acid to the developing mixture, finding that basic pH reduces CPM tailing and improves LCF peak shape. So, the amount of ammonia was optimized to get a final composition of toluene: ethanol: acetone: ammonia in a ratio (3:3:3:1 by volume). At 230 nm, densitometric detection was carried out and Rf values were 0.37 and 0.78 for CPM and LCF, respectively Fig. 4.

### Method validation

Spectrophotometric method (extraction technique, which included ICE, Z°E, US) and HPTLC-densitometric methods were validated according to the recent ICH guidelines [27, 28] to evaluate linearity, sensitivity, specificity, accuracy and precision.

The linearity ranges for both drugs were applied within 3.0–30.0 μg mL^**−**1^ for the proposed spectrophotometric method under the above-described experimental conditions. The validation parameters were summarised in Table 1. For HPTLC method, linear calibration curves between peak areas and corresponding drug concentrations were obtained within concentration ranges of 0.3–4.0 μg/spot and 0.8–10.0 μg/spot for CPM and LCF, respectively (Fig. 4). Table 1 shows the regression equations and regression parameters that were obtained.

**Table 1:** Analytical performance data for the determination of the studied drugs by the proposed spectrophotometric and HPTLC-densitometric methods

Parameters	ICE	Z°E/US	TLC-densitometric	
	CPM	LCF	CPM	LCF
Linearity range	3.0–30.0 μg mL^−1^	3.0–30.0 μg mL^−1^	0.3–4.0 μg/spot	0.8–10.0 μg/spot
Regression equation				
Slope ± SD	1.2393 ± 0.00003	0.0397 ± 0.00019	3604.31 ± 13.95	3078.49 ± 20.19
Intercept ± confidence interval^*^	0.0017 ± 0.0006	0.0076 ± 0.003	743.28 ± 26.334	122.46 ± 19.293
Correlation Coefficient (r)	0.999	0.999	0.999	0.999
LOD	0.13 μg mL^−1^	0.66 μg mL^−1^	0.03	0.13
LOQ	0.40 μg mL^−1^	2.00 μg mL^−1^	0.09 μg/spot	0.40 μg/spot

^*^ $\overline{x}\pm t\frac{s}{\sqrt{n} }$ where $\overline{x }$ is the mean value, and *s* is the standard deviation at *n* = 8 and the statistic value of t is 2.365 at 95% confidence interval with 7 degrees of freedom

The proposed HPTLC method’s sensitivity was evaluated and stated based on LOD (3.3 SD/S) and LOQ (10 SD/S), where SD is the standard deviation of multiple blank samples and S is the slope of the drug calibration curve, and the results are shown in Table 1. Meanwhile, for the novel ICE technique, LOQ was determined according to the ICH recent guidelines [29] based on visual evaluation. The concentrations of LCF and CPM were prepared in dilutions at lower concentration s below the lower concentration range. The ICE method was used to calculate each drug’s concentration to the minimum level at which the analyte was resolved and quantified reliably. Table 1 shows also the LOQ as obtained practically for both drugs using the proposed novel ICE technique.

The proposed methods’ accuracy was investigated by comparing the results to the reported method [16]. According to Statistical analysis of the data [30], there was no significant difference in accuracy between them (Table 2). Table 3 shows the results of tests on repeatability and intermediate precision. The RSD% values were found to be less than 2 units, confirming the proposed method’s high precision.

**Table 2:** Application of the proposed spectrophotometric and HPTLC-densitometric methods for the determination of the studied drugs in pure form

Drug	UV–Vis Spectrophotometry			TLC-densitometry			Reported method [16]		
	Conc. added (μg mL^−1^)	Found^*^ (μg mL^−1^)	%Recovery	Conc. added (μg/spot)	Found^*^ (μg/spot)	%Recovery	Taken (μg mL^−1^)	Found^*^ (μg mL^−1^)	%Recovery
CPM	6	5.96	99.33	0.3	0.30	102.40	10	10.04	100.44
	8	8.13	101.63	0.7	0.69	98.37	20	19.91	99.56
	12	11.98	99.83	1	1.01	100.91	30	30.04	100.15
	16	16.09	100.56	2	1.99	99.98			
	22	21.91	99.59	3	2.98	99.48			
	28	28.31	101.11	4	4.01	100.28			
Mean% ± SD			100.34 ± 0.90			100.24 ± 1.36			100.05 ± 0.45
*t*-test	0.51 (2.36)			0.22 (2.36)					
LCF	6	6.04	100.67	0.8	0.79	98.56	10	9.86	98.63
	8	7.93	99.13	2	2.01	100.25	20	20.27	101.36
	12	12.26	102.17	4	4.00	100.11	30	29.86	99.55
	16	15.89	99.31	6	5.97	99.46			
	22	22.18	100.82	8	8.08	101.05			
	28	27.87	99.54	10	9.95	99.50			
Mean% ± SD			100.27 ± 1.16			99.82 ± 0.85			99.85 ± 1.39
*t*-test	0.40(2.36)			0.04 (2.36)					

^*^ Comparison method involved HPLC determination of CPM and LCF using C18 column with mobile phase of buffer (pH 6.5) and acetonitrile (50:50, % v/v) as isocratic mobile phase, run time 8 min, flow rate 1 mL/min,linearity ranges 2–6 μg mL^−1^ and 10–30 μg mL^−1^ for CPM and LCF respectively and UV detection at 227 nm

**Table 3:** Precision data for the determination of the studied drugs by the proposed spectrophotometric and HPTLC-densitometric methods

Drug	Method		Intra-day^a^				Inter-day^b^			
	UV–Vis method	HPTLC-densitometry	UV–Vis method		HPTLC- Densitometric		UV–Vis method		HPTLC-densitometric method	
	Taken (μg mL^−1^)	Taken (μg/ spot)	Mean ± SD^*^	Precision (RSD%)	Mean ± SD^*^	Precision (RSD%)	Mean ± SD^*^	Precision (RSD%)	Mean ± SD^*^	Precision (RSD%)
CPM	5.0	0.3	100.02 ± 0.41	0.42	101.87 ± 0.23	0.23	100.13 ± 0.12	0.11	101.97 ± 1.63	1.60
	15.0	2.0	100.26 ± 0.55	0.55	99.41 ± 0.97	0.98	100.28 ± 0.04	0.04	99.65** ± **1.02	1.02
	30.0	4.0	100.30 ± 0.51	0.50	99.96 ± 1.26	1.26	100.42 ± 0.05	0.05	98.85** ± **0.84	0.85
LCF	5.0	0.8	100.17 ± 0.47	0.47	101.59 ± 0.54	0.53	100.07 ± 0.41	0.41	101.29 ± 1.35	1.33
	15.0	5.0	99.98 ± 0.37	0.37	99.83 ± 1.52	1.52	99.65 ± 0.25	0.24	100.74 ± 1. 12	1.11
	30.0	10.0	100.21 ± 0.55	0.55	100.18 ± 0.73	0.73	100.33 ± 0.09	0.08	101.82 ± 1.07	1.05

^*^Average recovery results of three determinations; ^a^ within the day; ^b^ within three consecutive days using the proposed UV–Vis and HPTLC methods

The selectivity of the proposed ICE method was evaluated by the analysis of different laboratory prepared mixtures of CPM and LCF containing different ratios within the linearity range and good results were obtained and summarized in Table 4. The proposed method's validity was further evaluated by using the standard addition technique and calculating the concentrations of standard added. Table 5 summarizes the acceptable results that were obtained.

**Table 4:** Determination of CPM with LCF in laboratory prepared mixtures by the proposed spectrophotometric method

Ratio CPM:LCF	UV–Vis spectrophotometric method				TLC method			
	Laboratory prepared mixtures (μg mL^−1^)		Recovery%*		Laboratory prepared mixtures (μg mL^−1^)		Recovery%*	
	CPM	LCF	CPM	LCF	CPM	LCF	CPM	LCF
1:2	5	10	98.12	102.08	4	8	99.15	98.12
1:1	20	20	101.98	98.31	2	2	100.3	101.41
3:1	30	10	101.71	98.96	3	1	99.66	99.55
2:1	20	10	100.66	101.18	4	2	100.8	98.68
5:1	25	5	101.09	99.23	4	0.8	100.71	99.74
4:1	20	5	102.31	100.48	4	1	98.97	100.2
1:5	5	25	100.05	100.93	2	10	99.21	101.11
1:3	5	15	99.87	99.45	1	3	100.23	99.02
	Mean% ± SD		100.72 ± 1.37	100.07 ± 1.29	Mean% ± SD		99.88 ± 0.73	99.73 ± 1.15

^*^Average of three separate determinations

**Table 5:** Application of standard addition technique for the determination of CPM in combined Lupituss CPM® syrup formulations with LCF by the proposed TLC- densitometric and spectrophotometric methods

CPM				LCF			
Mean% ± RSD	Standard addition			Mean% ± RSD	Standard addition		
	Taken μg mL^−1^	Added μg mL^−1^	Recovery % of added		Taken μg mL^−1^	Added μg mL^−1^	Recovery % of added
Spectrophotometric method							
99.08 ± 0.75	10.0	5.0 10.0 15.0	102.32 100.15 99.56	100.31 ± 0.64	10.0	5.0 10.0 15.0	101.18 99.47 99.84
	Mean ± RSD%		100.68 ± 1.45		Mean ± RSD%		100.16 ± 0.90
HPTLC-densitometry method							
99.55 ± 1.93	1.0	0.3 2.0 3.0	101.33 98.89 100.49	99.68 ± 0.84	2.0	0.8 6.0 8.0	99.33 98.32 101.75
	Mean % ± RSD		100.24 ± 1.24		Mean % ± RSD		99.82 ± 1.76

Regarding the developed HPTLC method, the capacity factor (Kʹ), number of theoretical plates (N), resolution (Rs), tailing factor, and selectivity factor (α) were calculated to ensure system performance based on United States Pharmacopeia (USP) guidelines [31] before or during the analysis, and the system was found to be suitable (Table 6).

**Table 6:** Parameters of system suitability test of the HPTLC-densitometric method

Parameter	CPM	LCF	Reference value
Retardation factor (R_f_)	0.37	0.78	Less than 1
Capacity factor (K)	1.70	0.28	The higher the value longer the retention factor
Resolution (R_s_)	2.87		> 1
Tailing factor	0.92	0.8	= 1 for typical symmetric peak
Selectivity factor (α)	6.07		> 1

### Application to pharmaceutical formulations

The proposed methods were used to determine CPM and LCF in Lupituss-CPM® syrup. There was no interference from additives or excipients observed in the results. As shown in Table 7, the calculated *t*-test values are less than the tabulated ones, indicating no significant difference between the reported [16] and proposed methods, confirming accuracy and precision at 95% confidence limit.

**Table 7:** Assay results for the determination of CPM in combined dosage forms with LCF by the proposed spectrophotometric, HPTLC-densitometric and reference [16] methods

Dosage form	Spectrophotometric method				TLC- Densitometric method				Reported method [16]			
	Conc. taken (μg mL^−1^)		% Found		Conc. taken (μg/spot)		% Found		Conc. taken (μg mL^−1^)		% Found	
Lupituss CPM® syrup	CPM	LCF	CPM	LCF	CPM	LCF	CPM	LCF	CPM	LCF	CPM	LCF
	3.0	15.0	98.89	101.02	0.3	1.5	101.77	100.44	2.0	10.0	101.38	99.58
	4.0	20.0	99.92	100.16	1.0	5.0	98.25	99.83	5.0	25.0	98.24	99.56
	5.0	25.0	98.45	99.76	2.0	10.0	98.63	98.78	6.0	30.0	99.83	100.78
Mean ± SD			99.08 ± 0.75	100.31 ± 0.64			99.55 ± 1.93	99.68 ± 0.84			99.82 ± 1.57	99.97 ± 0.71
*t*-test			0.73 (2.776)	0.62 (2.776)			0.19 (2.776)	0.46 (2.776)				

In conclusion, the ICE method employs an innovative extraction strategy that allows for independent determination of the target compound’s concentration, overcoming matrix interferences and providing accurate measurements. By effectively separating the target compound from the complex matrix, ICE eliminates potential biases and enhances the robustness of the analysis. The method can be generalized to other mixtures in different overlay scenarios as proved by its advantages over the other classical methods.

### Evaluating the sustainability of the proposed method

The concept of green analytical chemistry is critical for the environment because it is urgent to achieve a high degree of greenness by reducing or eliminating hazards associated with chemical processes. The assessment of the greenness of analytical methods has recently gained motivation using various recent tools.

The green analytical procedure index (GAPI) is a recent tool for assessing the green character of the entire analytical procedure, from sample collection to sample preservation, transport, preparation, and finally determination in a pictogram composed of 15 zones [17]. A chart comprising the enumerated 15 zones is illustrated in supplementary materials Fig. S1 to clarify the assessment. GAPI evaluates the ecological impacts using a pictogram with five colored pentagrams representing each step of a procedure; there are three levels of color; green for low, yellow for medium, and red for high environmental impact. In general, the greater the number of steps involved in the procedure, the lower the greenness because energy consumption and waste volume will increase. For the first sample collection step, which is the time lag between sampling and determination. AGREE is another highly cited metric for evaluation that uses a numeric score. AGREE uses the same color code as GAPI, but employing the twelve principles of green analytical chemistry [18]. The green assessment profiles for the proposed methods compared to previously reported RP-HPLC method [16] on GAPI and AGREE are shown in Table 8. As revealed, GAPI pictograms show the best ecological impact for, the proposed spectrophotometric approach, then the proposed HPTLC, and the lowest is represented for the reference RP-HPLC method [16], as indicated by the number of red zones compared to green and yellow color codes. When AGREE pictograms are studied (Table 8; Supplementary materials table S2), the proposed spectroscopic approach suggested having the highest greenness score. Although the proposed HPTLC method have slightly better score than the reference HPLC method [16], however, in this situation we can clarify the cons of AGREE assessment compared to GAPI. AGREE metric doesn’t consider the waste treatment and recycling scoring compared to GAPI, where the proposed HPTLC method can recycle the mobile phase for several chromatographic runs compared to HPLC technique. This can be shown by the lower right pictogram color code for GAPI pictograms, where only the proposed HPTLC method can show green color for waste treatment compared to the proposed spectroscopic approach (yellow) and the reference method (red). The assessment tools indicate that the proposed methods can be better used for routine analysis of the studied mixture without causing environmental harm.

**Table 8:** Assessment of the proposed and reported methods for determination of CPM and LCF

Method	White analytical chemistry	GAPI	AGREE
Proposed HPTLC method			
Proposed spectroscopic method			
Reported HPLC method [16]			

White analytical chemistry (WAC) metric is a more recent tool which was created to assess analytical procedures from a variety of aspects [19], including efficiency, ecological impact, and cost-effectiveness. Numerous approaches exist for determining the long-term viability of a project, each with their own advantages and disadvantages. However, one strategy that has shown constant success is to employ several diverse strategies simultaneously. By combining the concepts of red, green, and blue, we may analyze the procedure's sustainability and arrive at a white result. As the red color indicates, the analysis was effective. Analytical data on red parameters contains information such as range of use, detection and quantification limits, accuracy, and precision. The twelve principles of green analytical chemistry (GAC) address the issues of reagent toxicity, reagent quantity, waste, energy use, and direct consequences. The blue represents input that relates to needs, requirements, and ease of operation. In a downloadable Excel spreadsheet, we give a table with three columns (red, green, and blue) to let you compare the long-term viability of two different approaches to analysis using the WAC metric. Based on the information provided, a score out of 100 is determined, which quantifies the whiteness of the analysis. This chart shows the percentage of each color and their merged white result. Scanning enables us to learn and analyze the effectiveness and whiteness of the analytical procedure being utilized. A high percentage of each color, but especially white, should result from the most efficient and sustainable analytical process.

A comparison of the published RP-HPLC [16] results with the proposed methods utilizing the WAC metric (Table 8; Supplementary materials document 2) reveals inconsistencies in the three-color parameters. The proposed spectrophotometric and HPTLC methods demonstrated superior performance compared to the reported HPLC method in terms of measuring red color, achieving scores of 105.0% and 110.0% versus 97.5%, respectively. This can be attributed to their heightened sensitivity, as evidenced by their lower limits of detection and quantification. Similarly, for green color, the proposed spectrophotometric and HPTLC methods outperformed the reported HPLC method, achieving scores of 96.3% and 87.1% versus 78.3%, respectively. This can be attributed to the reduced consumption of solvent, energy, and waste associated with the proposed methods. Furthermore, in terms of blue color, the proposed spectrophotometric and HPTLC methods also outperformed the reported HPLC method, achieving scores of 81.5% and 79.6% versus 75.6%, respectively. The proposed spectrophotometric and thin-layer chromatography (TLC) methods exhibit whiteness scores of 94.2% and 92.2% respectively, indicating a higher level of environmental friendliness compared to the reported method with a score of 83.8% as shown in Table 8.

Finally, the two proposed analytical techniques have been established and validated for the aimed purpose. The choice between the novel ICE/UV–Vis spectroscopy and HPTLC methods for the analysis of the drugs under study depends on the specific requirements of the analysis and availability of tools. Both techniques have their advantages and limitations. UV spectroscopy is widely used as a relatively simple and quick technique. Meanwhile, HPTLC is a separation technique that requires minimal sample preparation and is particularly useful for low concentrations of the analytes. However, it’s worth noting that the ecological impact of ICE tool is lower than that of the HPTLC methodology. Sometimes it may be beneficial to use both techniques in combination to complement each other’s strengths and/or limitations.

## Conclusion

According to the obtained results, it could be concluded that the suggested analytical techniques demonstrated selective and sensitive detection for both Chlorpheniramine maleate and Levocloperastine fendizoate either in their pure form or in combination. The spectrophotometric platform covered a wider linearity range while the chromatographic one showed more sensitivity. It is worth mentioning that the proposed ICE spectrophotometric method is feasible to be adopted for the analysis of complex binary and multi-component mixtures. In addition, the newly adopted methods have the privilege of being based on the extraction of the parent zero-order spectra of the cited drugs and measuring the concentrations at their λmax with maximum sensitivity and less effort during analysis. Both techniques are simple, accurate, precise and ecofriendly. Although toluene had been used in the developed HPTLC method, it was used at minor amounts without greatly affecting the method’s greenness as showed by the greenness assessment, and hence rendering the developed HPTLC method of lower greenness compared to the developed spectrophotometric method. In addition, both techniques are cost effective where they only require a spectrophotometer with a built-in software for spectral manipulation and a densitometer with no need for any sophisticated programs. The suggested methods could be applied in any quality control lab for checking and fast track the concentration change and/or degradation of drugs under study during any production stage, packaging and on shelves to assure safety and effectiveness of the marketed dosage forms.

### Supplementary Information

Supplementary Material 1.

Supplementary Material 2.

## Data Availability

No datasets were generated or analysed during the current study.
