# Supplementary material for: Independent concentration extraction as a novel green approach resolving overlapped UV–Vis binary spectra and HPTLC-densitometric methods for concurrent determination of levocloperastine and chlorpheniramine
Source: BMC Chem. 2024 Aug 28;18(1):160. doi: 10.1186/s13065-024-01260-w (PMC11360872; doi:10.1186/s13065-024-01260-w)
Supplement: Supplementary file 1 — Supplementary Material 1. [file 13065_2024_1260_MOESM1_ESM.docx]

**Supplementary materials**

**Independent Concentration Extraction as a Novel Green Approach Resolving Overlapped UV-Vis Binary Spectra and HPTLC-Densitometric Methods for concurrent Determination of Levoclopersatine and Chlorpheniramine**

Ekram H. Mohamed^1^, Hany A. Batakoushy^2^*, Adel Ehab Ibrahim^3,4^, Zeinab Adel Nasr^5^*, Marwa M. Soliman ^5^, Sona S. Barghash ^5^, Tahany F. Mohamed ^5^, and Fatma A. Fouad ^5^

^1^ Pharmaceutical Analytical Chemistry Department, Faculty of Pharmacy, The British University in Egypt, 11837, El sherouk city, Egypt; [Ekram.hany@bue.edu.eg](mailto:Ekram.hany@bue.edu.eg)

^2^ Pharmaceutical Analytical Chemistry Department, Faculty of Pharmacy, Menoufia University, Shebin Elkom, 32511, Egypt**;** [hany.batakoushy@phrm.menofia.edu.eg](mailto:hany.batakoushy@phrm.menofia.edu.eg)

^3^ Pharmaceutical Analytical Chemistry Department, Faculty of Pharmacy, Port-Said University, Port Said 42511, Egypt; adel.ehab@pharm.psu.edu.eg

^4^ Natural and Medical Sciences Research Center, University of Nizwa, P.O. Box 33, Birkat Al Mauz, Nizwa 616, Oman; pharmacist_adel_2005@yahoo.com

^5^ Pharmaceutical Analytical Chemistry Department, Faculty of Pharmacy (Girls), Al-Azhar University, Cairo 11754, Egypt; [zeinabadel@azhar.edu.eg](mailto:zeinabadel@azhar.edu.eg); [marwasoliman@azhar.edu.eg](mailto:marwasoliman@azhar.edu.eg); [SonaMohamed1926.el@azhar.edu.eg](mailto:SonaMohamed1926.el@azhar.edu.eg); (tahany.fouad@gmail.com, Tahanyosman.52@azhar.edu.eg); [fatmafouad2304.el@azhar.edu.eg](mailto:fatmafouad2304.el@azhar.edu.eg)

***** Correspondence: hany.batakoushy@phrm.menofia.edu.eg; [zeinabadel@azhar.edu.eg](mailto:zeinabadel@azhar.edu.eg)


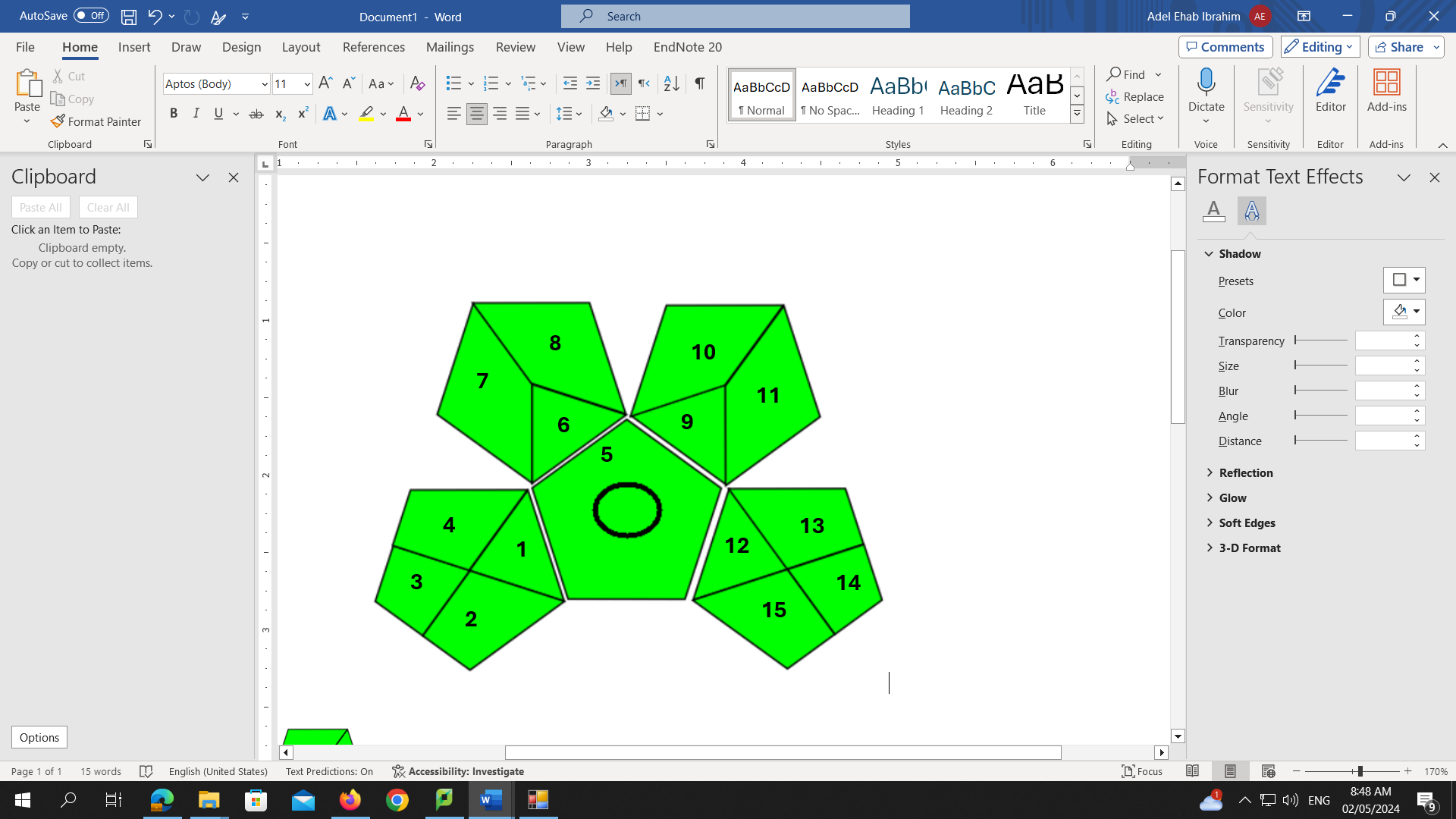


**Supplementary Fig. S1**: GAPI pictogram showing 15 zones representing different steps within the analytical procedure

(**Sample collection & preparation:** 1. Sample collection, 2. Preservation, 3. Transport, 4. Storage, 5. Method type, 6. Extraction scale, 7. Consumed reagents, 8. Extra treatment; **Solvents and reagents:** 9. Amount, 10. Health hazards, 11. Safety hazards; **Instrumentation:** 12. Energy, 13. Occupational hazards, 14. Waste, 15. Waste treatment)

**Supplementary Table S2**: AGREE assessment tool report

| **Green Analytical Chemistry Concenpt** | **Proposed HPTLC method** | **Proposed ICE tool** | **Reported method (16)** |
| --- | --- | --- | --- |
| 1. Sampling procedure | Off-line analysis | Off-line analysis | Off-line analysis |
| 1. Sample size | 0.000001 gm | 0.000010 gm | 0.000002 gm |
| 1. Analytical device positioning | Off-line | Off-line | Off-line |
| 1. Number of major steps in sample preparation | 3 or fewer | 3 or fewer | 3 or fewer |
| 1. Degree of automation:   Sample preparation: | Semi-automatic  None or miniaturized | Semi-automatic  None or miniaturized | Automatic  None or miniaturized |
| 1. Derivatization | None | None | None |
| 1. Waste amount | 1 gm | 2 mL | 8 mL |
| 1. Number of analytes per run:   Sample throughput: | 2 analytes  21 samples | 2 analytes  12 samples | 2 analytes  7 samples |
| 1. Energy | UV-Vis spectroscopy | UV-Vis spectroscopy | LC |
| 1. Type of reagents | Some reagents are bio-based | Some reagents are bio-based | Some reagents are bio-based |
| 1. Involving toxic reagents:   Amounts: | Yes  90 mL | No  -- | Yes  4 mL |
| 1. Threats | - Toxic to aquatic life - Biocumulative - Highly flammable | - Highly flammable | - Toxic to aquatic life - Biocumulative - Persistant - Highly flammable |
